# Efficient removal of Cr(iii) by microbially induced calcium carbonate precipitation

**DOI:** 10.1039/d4ra05829a

**Published:** 2025-01-29

**Authors:** Jia Qin, Huan Cao, Yang Xu, Fei He, Fengji Zhang, Wenqiang Wang

**Affiliations:** a College of optoelectronic manufacturing, Zhejiang Industry and Trade Vocational College Wenzhou 325002 China Qinjia@zjitc.edu.cn; b School of Materials Science and Engineering, Lanzhou University of Technology Lanzhou Gansu 730050 China; c China Railway Heavy Machinery Co. Ltd Wuhan 430077 China

## Abstract

Microbially induced calcium carbonate precipitation (MICP) has emerged as a promising technique for environmental remediation, particularly for heavy metal removal. This study explores the potential of MICP for Cr(iii) removal, analyzing the effects of temperature, pH, calcium source addition, and initial Cr(iii) concentration on removal efficiency. The results show that Cr(iii) can be efficiently removed with a removal rate approaching 100% under optimal conditions (25 °C, pH 7.0, 1.0 g CaCl_2_). The presence of Cr(iii) induces the transformation of CaCO_3_ crystals from calcite to spherulitic aragonite, forming Cr-bearing carbonate compounds and hydroxides. This study provides insights into the mechanisms and optimal conditions for MICP-mediated Cr(iii) removal, highlighting its feasibility and effectiveness for large-scale environmental remediation and offering an economical and environmentally friendly solution to Cr contamination.

## Abstract 2

Removal of Cr(iii) by microbially induced calcium carbonate precipitation.

## Graphical abstract

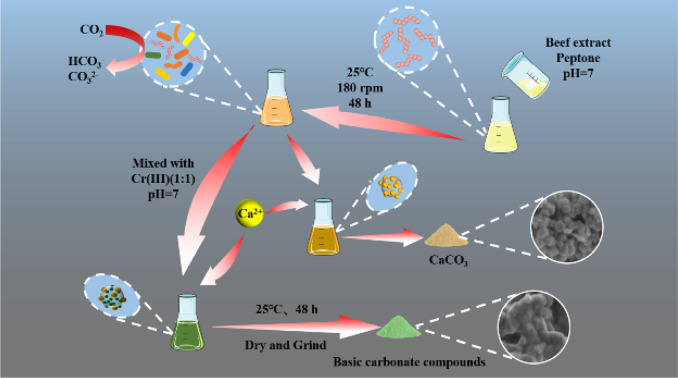

## 1 Introduction

Environmental pollution caused by heavy metals in water has become an urgent global problem that poses a major threat to human health, biodiversity, and ecological stability.^1^ Among these pollutants, chromium has caused widespread environmental pollution owing to its extensive use in processes such as leather tanning, metal plating, dye manufacturing, and automobile production.^2^ The dual oxidation states of Cr(iii) and Cr(vi) complicate their impact on the environment, with Cr(vi) being considerably more toxic than Cr(iii) owing to its carcinogenicity and mobility across ecosystems.^3^ Cr(vi) has been extensively studied.^4^ Traditional Cr remediation methods include chemical precipitation, adsorption, ion exchange, and membrane filtration. These methods are often limited in terms of efficiency, sustainability, and economic viability, particularly when dealing with large areas with low pollutant concentrations.^5^ The secondary waste generated by these processes complicates the environment in which they are applied, highlighting the need for innovative solutions that are effective and environmentally friendly. Therefore, bioremediation is a promising approach.^6^ According to previous studies, most bioremediation methods remove Cr(vi) only by reducing it to Cr(iii).^7^ The main form of Cr present in pollutants, such as in the tannery industry, is Cr(iii). Cr(iii) compounds, such as CrF_3_, have a wide range of industrial applications, particularly as catalysts. Cr(iii) is less toxic to the human body and is beneficial in trace amounts, excessive intake can still lead to chromium poisoning, posing a threat to human health. Cr(iii) can be oxidized to the more toxic Cr(vi) under certain environmental conditions, such as high temperature or extreme pH levels.^8^ Cr(vi) is more harmful due to its carcinogenic properties and mobility across ecosystems, which increases the risk to both the environment and living organisms. Cr(iii) is the predominant form of chromium in many industrial pollutants, such as in the leather tanning industry, it is essential to remove it effectively. By addressing Cr(iii), we can not only prevent its further oxidation to Cr(vi) in the environment but also mitigate potential health risks. In pursuit of ecological friendliness and economic efficiency, developing bioremediation methods capable of effectively removing Cr(iii) from water is crucial.^9^

Microbially induced calcium carbonate precipitation (MICP) offers significant advantages over other conventional bioremediation methods, such as biosorption, bioreduction, and bioaccumulation, in heavy metal removal. Unlike biosorption, which relies on the physical adsorption of metal ions onto biomass.^10^ MICP precipitates metal ions as insoluble carbonates through microbial metabolism, reducing the risk of secondary pollution. Unlike bioreduction methods that primarily reduce Cr(vi) to Cr(iii), MICP not only removes Cr(iii) but also eliminates Cr(vi) through carbonate mineralization, providing a more comprehensive solution. In contrast to bioaccumulation, which depends on the absorption and storage of metals by microorganisms, MICP not only removes heavy metals from the environment but also converts them into more stable and non-toxic forms. Metal carbonates, such as chromium carbonate, precipitated by MICP are less likely to re-enter the ecosystem, offering a long-term solution for heavy metal pollution control. Utilizing microorganisms with minimal chemical inputs, MICP is a more cost-effective and environmentally friendly alternative method, suitable for large-scale applications.^11^ MICP is a common bio-induced mineralization reaction that catalyzes the formation of CaCO_3_ precipitation through the natural biochemistry of microorganisms. CaCO_3_ can remove heavy metal ions through binding or coprecipitation.^12^ Microorganisms capable of inducing carbonate precipitation include urease-producing, carbonic anhydrase (CA)-producing, and sulfate-reducing microorganisms, with urease and CA being the two most widely used enzymes.^13^ Understanding the mechanism of enzyme action is essential for optimizing the removal of heavy metals by MICP. Urease catalyzes the hydrolysis of urea, producing NH_3_ and CO_2_. The NH_3_ increases the pH, promoting the precipitation of heavy metals as hydroxides or carbonates, which aids in their removal. Carbonic anhydrase (CA) catalyzes the conversion of CO_2_ and H_2_O to bicarbonate, speeding up CO_2_ hydration by a factor of 10^7^.^14^ This reaction helps regulate pH, essential for metal carbonate precipitation. The bicarbonate formed reacts with metal ions to precipitate insoluble metal carbonates.CA offers advantages over urease, including higher catalytic efficiency and lower energy requirements, making it more suitable for large-scale applications.^15^ Unlike urease, which significantly raises the pH, CA maintains a more balanced pH, reducing unwanted byproducts.^16^ Urease-producing microorganisms generate large amounts of ammonia–nitrogen byproducts, and excess ammonia–nitrogen in untreated wastewater can acidify water quality and promote eutrophication.^17^ CA promotes the conversion of CO_2_ to bicarbonate and carbonate, effectively fixing and reducing atmospheric CO_2_, which suggests new ideas for the global carbon cycle and climate change.^18^ In summary, although CA and urease play unique roles in the MICP process, the efficiency, speed, and environmental compatibility of CA provide clear advantages for heavy metal removal. Therefore, the use of CA in the MICP process is considered more environmentally friendly and sustainable. The ability of CA to promote the selective precipitation of metal carbonates offers a targeted approach for heavy metal removal, allowing for the controlled and efficient precipitation of heavy metals,^19^ making it an attractive bioremediation strategy.

The application of MICP in Cr(iii) removal is promising but faces challenges. These include optimizing the conditions to maximize efficiency, understanding the interaction mechanisms between the precipitated CaCO_3_ and Cr(iii), and scaling up the process from a laboratory setting to field application. This biotechnological approach provides a more environmentally friendly and cost-effective solution to heavy metal pollution and offers a versatile process for environmental restoration.^20^ Reliance on natural microorganisms and their ability to operate under contaminated conditions enhance their attractiveness as sustainable remediation strategies.^21^ MICP for Cr(iii) removal is at the forefront of environmental science. In this study, we investigated the removal of Cr(iii) by MICP using a novel carbonate-mineralizing bacterium (CB) capable of capturing CO_2_ by secreting CA. The optimal growth environment for the strains and changes in pH over time were investigated. The effects of time, pH, temperature, Ca^2+^ dosage, initial Cr(iii) concentration, and other factors on the Cr(iii) removal were also examined. The mineralization products produced under different Cr(iii) concentrations were systematically analyzed, and the mineralization removal mechanism of Cr(iii) by this strain was explored. This biochemical pathway provides a gentle, natural, and effective method for the complete removal of toxic Cr(iii) from ecosystems, which is important for environmental clean-up and restoration.

## 2 Materials and methods

### 2.1 Chemicals and reagents

Beef extract and peptone were purchased from Shanghai Yuanye Biotechnology (China, microbiological culture grade); acetic acid *p*-nitrophenyl ester, diethylmalonic acid, *p*-nitrophenol, anhydrous ethanol, sodium dihydrogen phosphate, disodium hydrogen phosphate, hexahydrate chromium chloride, calcium chloride, and potassium bromide were purchased from Shanghai McLin Biochemical Technology (China, AR).

### 2.2 CB culture and growth status

In this study, a novel CB of the genus Bacillus, which is characterized by rapid reproduction, high vitality, and high CA activity, was used. The bacterial powder was added to a natural liquid medium containing 2.0 g of bacterial powder, 1 g of beef paste, 0.6 g of peptone, and 200 mL of ultrapure water. The mixture was activated and cultured in a THZ-98AB constant-temperature shaking incubator (Shanghai YIHENG Scientific Instrument, China) at 25 °C and 180 rpm for approximately 48 h, then stored at 4 °C.

To determine the growth status of CB and optimal incubation conditions, the absorbance at 600 nm was measured using a UV-1800PC-DS2 UV-visible spectrophotometer (Shanghai Mepda Instrument, China). According to Lambert's law, the OD_600_ value, which measures solution turbidity, is proportional to the number of bacteria.^22^ This value was used to indirectly indicate the growth of the CB. OD_600_ values were measured by diluting the bacterial liquid five times at intervals of 6, 12, 18, 24, 30, 36, 42, and 48 h. Pre-experimentation determined dilution times to ensure absorbance ranged between 0.2 and 0.8, enhancing data reliability.

The optimal pH for the liquid medium was determined by adjusting the pH to 5, 6, 7, 8, or 9 using a PHS-3E pH meter (Shanghai Yidian Scientific Instrument, China) and pre-configured with 1 M HCl and 1 M NaOH. This helped us to elucidate the metabolic processes of CB over time. To determine the appropriate amount of bacterial powder, the pH was set to 7 and one-factor variable experiments were conducted with bacterial powder amounts of 0.5, 1, 2, 3, and 4 g. OD_600_ values were measured at 24 h and 48 h to observe bacterial growth and determine the optimal amount of bacterial powder.

**Fig. 1:**
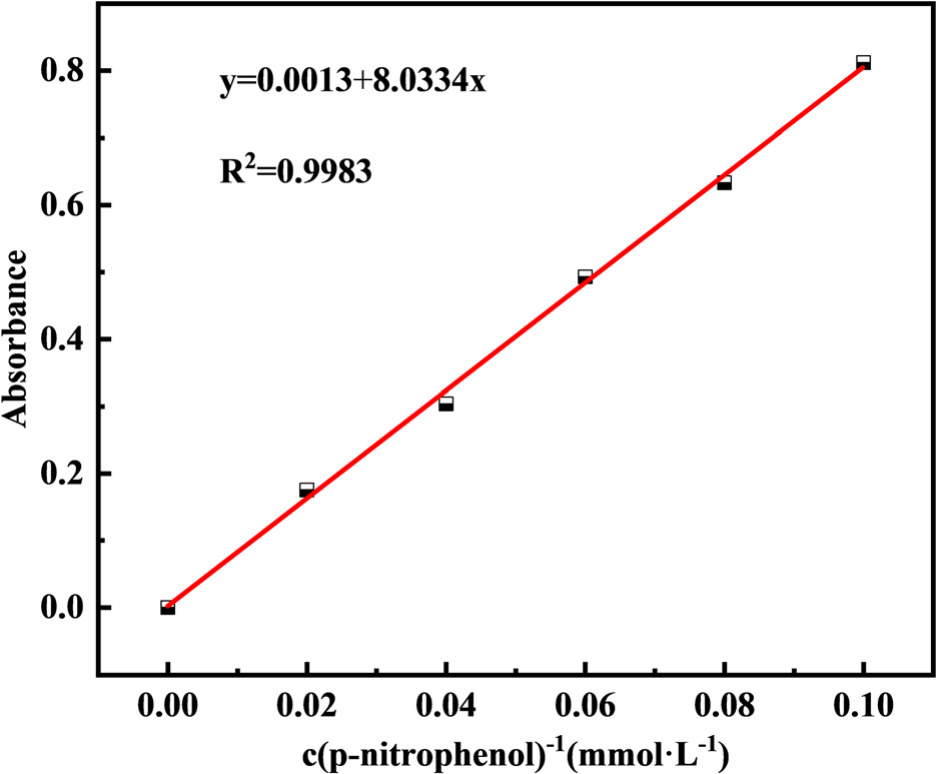
Standard curve of concentration and absorbance of *p*-nitrophenol.

Additionally, to monitor CB growth, the medium was adjusted to pH = 7, and 2 g of bacterial powder was added. The pH and CA activity of the bacterial solution were measured at 6 h intervals. CA activity was determined using a colorimetric method, which catalyzes the formation of *p*-nitrophenol from *p*-nitrophenyl acetate and diethylmalonic acid, reflecting enzyme activity based on the amount of *p*-nitrophenol formed.^23^ Phosphate buffer was used as the solvent to obtain *p*-nitrophenol concentrations of 0.02, 0.04, 0.06, 0.08, and 0.1 mM, and the standard absorbance curve at 405 nm is plotted in Fig. 1. The working solution was prepared by dissolving 0.0181 g of *p*-nitrophenyl acetate in 1 mL of anhydrous ethanol, and mixing it with 0.156 g of diethylmalonic acid fixed to 100 mL of phosphate buffer. CA activity was determined by taking 10 mL of the bacterial supernatant after centrifuging at 10 000 rpm for 5 min. Bacterial (0.1 mL) and working (0.1 mL) solutions were mixed in a 96-well enzyme labeling plate, incubated at 25 °C for 30 min, and the absorbance OD_405_ was measured at 405 nm. The amount of *p*-nitrophenol was determined by substituting the absorbance of a standard curve. A blank control was used to exclude interference from the self-hydrolysis of *p*-nitrophenyl lipids by acetic acid. CA activity was expressed in U, where 1 U represents 1 μmol of *p*-nitrophenol produced per minute.

All equipment was sterilized using an LDZX-75L-I vertical autoclave sterilizer (Shanghai Shen'an Medical Instrument Factory, China) for 30 min at 121 °C before the experiments. All experiments were conducted in duplicate to ensure the reliability of the results.

### 2.3 Cr(iii) removal experiment

The objectives of this experiment were to determine the Cr(iii) removal efficiency of CB *via* MICP under different initial Cr(iii) concentrations, temperatures, CaCl_2_ additions, and pH conditions, and to identify the optimal reaction time for Cr(iii) removal by CB. To investigate the optimal temperature for Cr(iii) removal, a 1000 mg L^−1^ Cr(iii) solution was prepared using CrCl_3_·6H_2_O. Subsequently, 100 mL of this solution was reacted with 100 mL of a bacterial solution (1 : 1) with the addition of 1.0 g of CaCl_2_. The pH was adjusted to 7 using 1 M HCl and 1 M NaOH, and the solution was incubated in an SPX-150 intelligent biochemical incubator (Shanghai YiXi Instrument and Equipment, China) at 20, 25, 30, 35, and 40 °C. The supernatant was collected every 3 h to determine the residual Cr(iii) concentration.

To investigate the removal efficiency at different initial Cr(iii) concentrations, Cr solutions of 100, 300, 500, 1000, 1500, 3000, and 6000 mg L^−1^ were prepared. Each solution was reacted with an equal volume of bacterial solution (1 : 1) containing 1 g of CaCl_2_, and the pH was adjusted to 7. The solutions were incubated at 25 °C, and the Cr(iii) concentration in the supernatant was measured after 48 h. To examine the effect of different CaCl_2_ additions on Cr(iii) removal, 1000 mg L^−1^ Cr(iii) solution was reacted with bacterial solution (1 : 1) with varying CaCl_2_ additions of 0, 0.5, 1.0, 1.5, and 2.0 g. The pH was adjusted to 7, and the solutions were incubated at 25 °C. The supernatant was sampled at 0, 3, 6, 12, 18, 24, 36, and 48 h to determine the residual Cr(iii) concentration. To investigate the effect of different pH levels on Cr(iii) removal, a 1000 mg L^−1^ Cr(iii) solution was reacted with bacterial solution (1 : 1) with 1.0 g of CaCl_2_, and the pH was adjusted to 3, 4, 5, 6, and 7. Acidic and neutral environments were selected to avoid interference from the alkaline precipitation of metal ions. The solutions were incubated at 25 °C, and the supernatant was sampled at 0, 3, 6, 12, 18, 24, 36, and 48 h to detect the remaining Cr(iii) concentration. Unless otherwise specified, the default culture conditions were 25 °C, 2.0 g of bacterial powder, initial pH = 7, and 48 h of incubation.

**Fig. 2:**
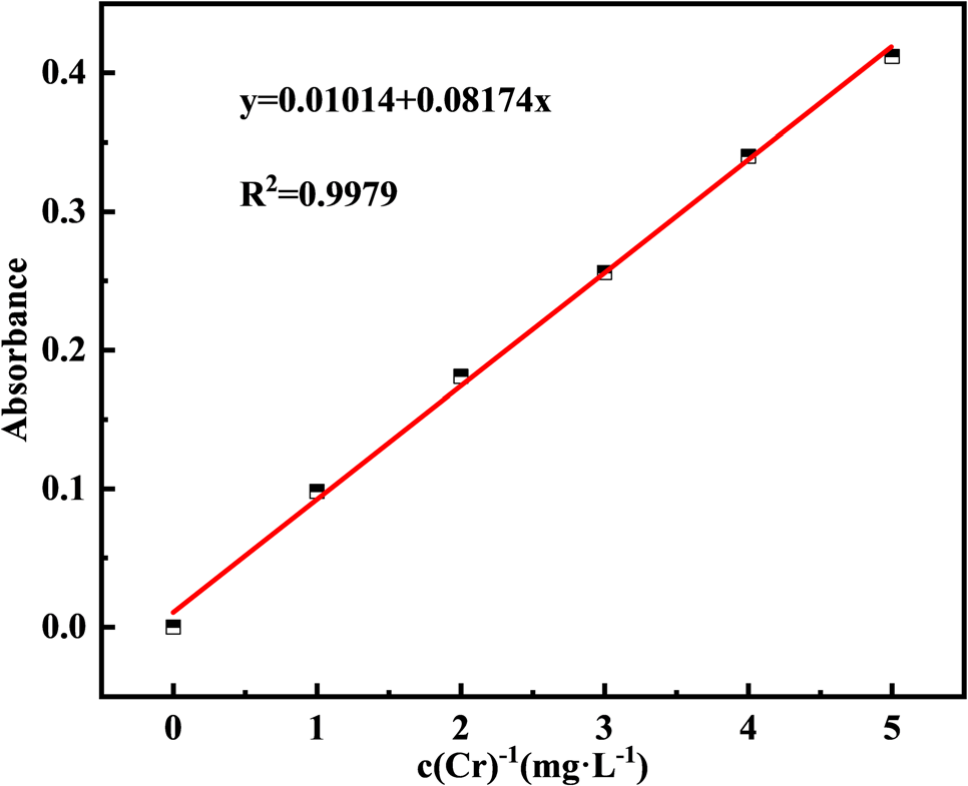
Standard curve of Cr and absorbance.

The Cr(iii) concentration in the test solution (*c*) was calculated by subtracting the Cr(vi) concentration (*c*_2_) from the total Cr concentration (*c*_1_). Atomic absorption spectrometry (AAS) at 357.9 nm was used to determine the total Cr concentration (*c*_1_),^24^ and 1,5-diphenylcarbazide (DPC) at 540 nm was used to detect the residual Cr(vi) concentration in the supernatant (*c*_2_).^25^ The negligible Cr(vi) content indicated that Cr(iii) was not converted to Cr(vi) in this study; therefore, the total Cr concentration measured is the Cr(iii) concentration (*c* = *c*_1_). Using the AAS method, a standard curve for total Cr was created by configuring standard solutions of 0, 1, 2, 3, 4, 5 mg L^−1^ and determining their absorbance (Fig. 2).

### 2.4 SEM and XRD

**Fig. 3:**
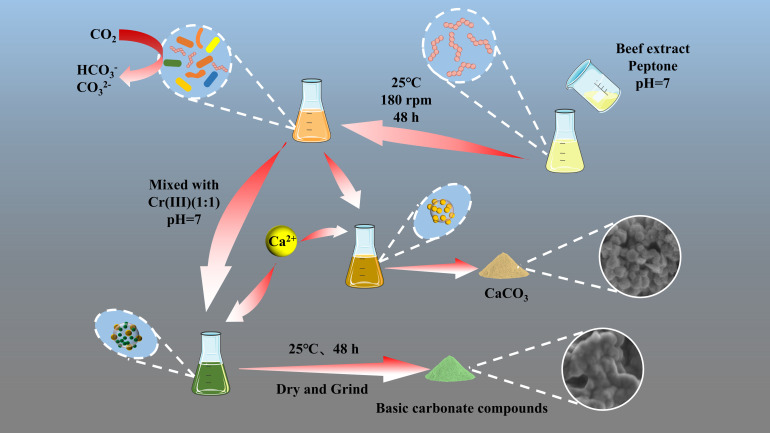
Schematic diagram of microbiologically induced calcium carbonate precipitation for removal of Cr(iii).

After adding the Cr(iii) solution and allowing it to react for 48 h, the supernatant was removed, and the remaining solution was centrifuged using an H1750R centrifuge (Hunan Xiangyi Laboratory Instrument Development, China) at 8000 rpm for 5 min. The precipitate was then rinsed three times with physiological saline and freeze-dried for 24 h using a Scientz-10YG Vacuum Freeze-Dryer (Ningbo Xinzhi Freeze-Drying Equipment, China). The dried samples were ground into powder using an agate mortar and pestle, bagged, and characterized within 48 h. The Cr(iii) removal experiments were conducted under the following conditions unless specified otherwise: temperature at 25 °C, 1.0 g of CaCl_2_, pH = 7, and a reaction time of 48 h. To determine the composition of the mineralization products, substrates formed by adding Cr(iii) at concentrations of 0, 500, 1000, 1500, and 3000 mg L^−1^ were analyzed. These samples were labeled CB + Ca + *n*Cr (*n* = 0, 500, 1000, 1500, and 3000) (Fig. 3).

The morphological characteristics of the substrates were observed using an SU8010 field emission scanning electron microscope (SEM, Hitachi, Tokyo, Japan) at a stable voltage of 3.0 kV. Energy-dispersive spectroscopy (EDS) was used to analyze the composition of the mineralized substrates. Because of the non-conductive nature of the substrates, a gold coating was applied using an EM ACE600 high-vacuum ion-sputtering instrument (Leica, Wetzlar, Germany). To enhance the clarity, the ground powder samples were mixed with alcohol, ultrasonically dispersed for 10 min, and a drop was placed on a 5 × 5 mm silicon wafer. After drying on conductive adhesive, the samples were gold-coated with a sputtering current of 35 mA for 80 s. To characterize precipitated mineralization products, a Bruker D8 ADVANCE X-ray diffractometer (XRD, Bruker, Karlsruhe, Germany) was used. The analysis employed a copper target, with a step size of 0.02°, scanning range from 5 to 80°, and a speed of 4° min^−1^. The XRD diffraction peaks were analyzed using the MDI Jade9 software.

### 2.5 FT-IR and XPS

To identify the chemical bonds and functional group structures in the generated substrates, the prepared samples were pressed and analyzed using an FTIR-650 Fourier-transform infrared spectrometer (FT-IR, Tianjin Gangdong Science and Technology, China). The molecular structures of the compounds were determined, and the data were exported and analyzed for functional group positions using OMNIC software.

Additionally, a small amount of the sample powder was analyzed to detect the valence states and chemical bonding of the elements on the sample surface using an ESCALAB 250XI X-ray photoelectron spectrometer (XPS, Thermo Fisher Scientific, MA, USA). The analysis chamber vacuum was set to 5 × 10^−10^ Pa, with an excitation source of Al Kα radiation (*hv* = 1486.68 eV), an operating voltage of 15 kV, and a filament current of 10 mA. The signal accumulated over 5–10 cycles. Data were then processed and peak-fitted using the Avantage software.

## 3 Results and discussion

### 3.1 The growth of the bacteria

**Fig. 4:**
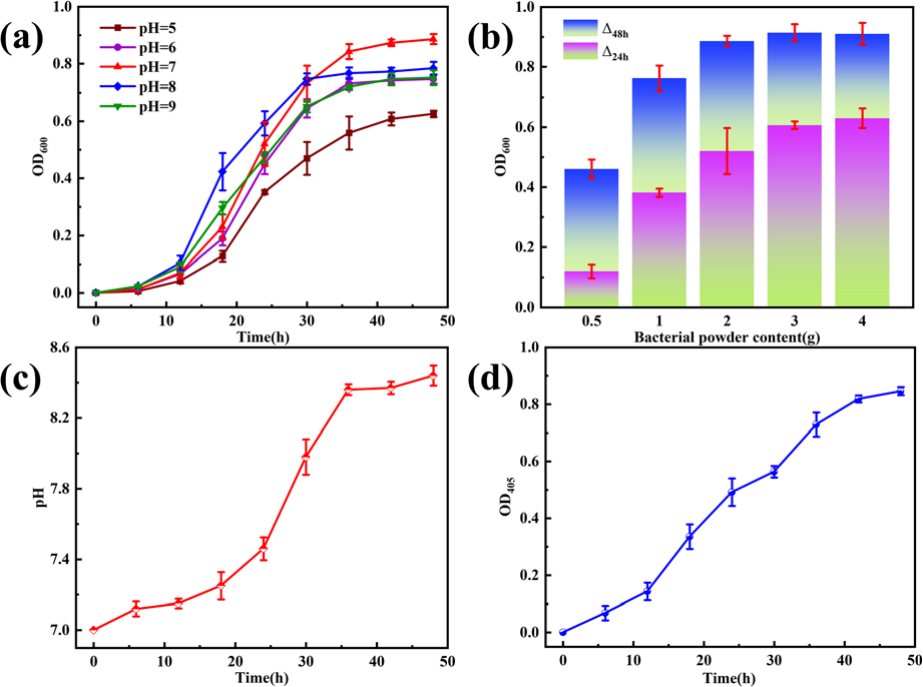
The change in the number of OD_600_ under different (a) initial pH and (b) bacterial powder additions. The change in CB growth process under (c) pH and (d) OD_405_.

OD_600_ was directly proportional to the amount of CB growth. Fig. 4a shows a plot of the OD_600_ change over time at different initial pH values of the culture solution. With increasing culture time, the number of bacteria continued to increase; 0–12 h is the flat period of bacterial growth, in which the bacteria from the dormant period began to enter the growth period; 12–36 h is the rapid growth stage of the bacteria; and 36–48 h is the stable period, in which the bacterial species began to reach a stable growth state, and the number of bacterial colony no longer increase and remain relatively stable. The initial pH of the culture medium also affected the growth condition of the bacteria, and the growth of the bacteria at pH 5–9 showed an overall trend of increasing and then decreasing; the species had the highest number of bacteria at pH = 7, indicating that it is more favorable for the growth of the bacteria in a neutral initial environment. During the rapid growth phase, the culture solution grew faster at pH > 7 than at pH 7; however, the final number of bacteria was still low. This suggests that an alkaline environment may be more favorable for the growth and reproduction of this species during the initial phase of rapid growth; however, too high a pH can inhibit bacterial reproduction (Fig. 4c). Growth curves at pH 9 were significantly lower than those at pH 8. As the growth and metabolism of the bacteria increased, the solution pH increased. Fig. 4c shows that the change curve of the initial pH of the culture solution is 7. This is due to the use of beef paste and peptone as nutrients in this study, both of which contain a large number of organic nitrogen compounds, which are decomposed by the microorganisms to produce alkaline substances such as amines, thus increasing the pH.^26^ The increase in pH and the presence of CO_2_ causes the substrate to form metal carbonates in addition to hydroxides. In summary, the bacteria used in this study had a culture solution with a pH of 7 and an incubation time of 48 h. Fig. 4b shows the increase in the OD_600_ corresponding to the addition of different bacterial powders. The OD_600_ showed an increasing trend as the bacterial powder continued to increase. The OD_600_ value peaked at 48 h with the addition amount of 3.0 g; however, the amount of change from 24 to 48 h was significantly lower compared with the addition amount of 2.0 g. This phenomenon suggests that the process of bacterial powder addition from 2.0 g to 3.0 g will inhibit bacterial growth; therefore, the bacterial powder addition of 2.0 g was used in this study. In Fig. 4d, the trend of CA activity and OD_600_ and pH change curves are basically the same, the enzyme activity increases with increasing time, and the highest activity reaches 6.67 U mg^−1^ at 48 h, indicating that the growth of CA is in good condition.^27^

### 3.2 Cr(iii) removal results

**Fig. 5:**
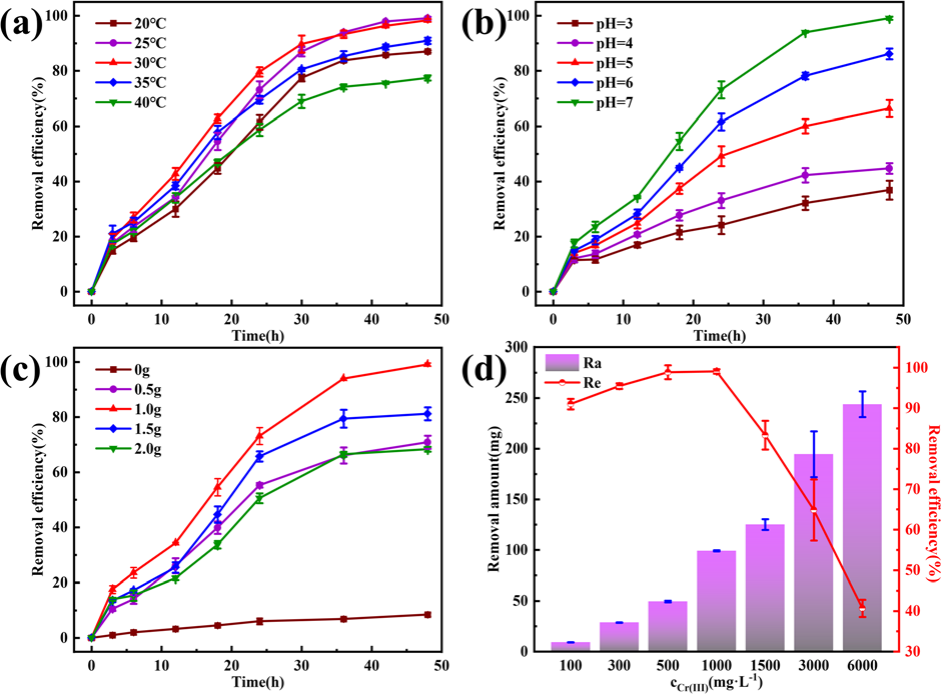
The removal effect of Cr(iii) by MICP under different conditions. (a) Temperature; (b) pH; (c) amount of CaCl_2_; (d) initial Cr(iii) concentration.

Temperature, pH, CaCl_2_ concentration, and initial Cr(iii) concentration are crucial factors affecting Cr(iii) removal *via* MICP.^28^ Temperature, in particular, affects the growth and metabolic activity of microorganisms by influencing the activity of biomolecules, such as enzymes and proteins, the fluidity of cell membranes, and the solubility of substances.^29^ Therefore, controlling the temperature is essential for optimizing microbial growth, as illustrated in Fig. 5a, where the Cr(iii) removal rate increased with reaction time and then gradually stabilized at different ambient temperatures. The removal efficiency was consistently high, >75%, within the temperature range of 20–40 °C. Specifically, Cr(iii) removal reached nearly 100% at 25 °C and 30 °C after 48 h of reaction, with efficiencies of 99.08% and 98.33%, respectively. In the early stages of the reaction, increasing the temperature from 20 °C to 30 °C resulted in a rapid increase in the Cr(iii) removal rate. This suggests that within a certain temperature range, a moderate increase in temperature supports microbial growth and propagation, thereby enhancing the production of CA, which is crucial for Cr(iii) removal. However, further temperature increases beyond 30 °C led to a decrease in the Cr(iii) removal rate, likely due to the denaturation of bioactive substances and other adverse changes that inhibit microbial growth. This is evident from the gradual decline in removal efficiency from 30 °C to 40 °C. To balance optimal microbial activity and energy consumption, 25 °C was selected as the reaction temperature for this study.

The pH significantly affects the growth and metabolic activity of microorganisms,^30^ thereby influencing the Cr(iii) removal rate. As shown in Fig. 5b, Cr(iii) removal increased with increasing pH, peaking at a pH of 7. This behavior, while different from that of many bacteria studied, aligns with consistent results in this research.^31^ In this study, the bacteria-produced CA facilitated the CO_2_ hydration reaction, producing CO_3_^2−^ and HCO_3_^−^ ions, which promoted Cr(iii) removal. As the pH increases, the concentration of H^+^ ions decreases, further enhancing the CO_2_ hydration reaction. In acidic solutions, CO_2_ and H_3_O^+^ ions compete with metal cations for active sites on the bacterial surface,^32^ inhibiting Cr(iii) adsorption, and the internal pH of most microbial cells is neutral. Thus, maintaining an external pH close to neutral helps preserve the structural stability of bioactive molecules and enzymes within the cells.^33^ Therefore, the pH of the reaction solution was adjusted to 7 to optimize Cr(iii) removal.

Calcium sources are crucial for MICP and serve as essential elements for microbial growth, maintaining cellular structural stability and ensuring osmotic pressure homeostasis. The addition of a calcium source also enhanced the mineralization process of MICP by providing binding sites for heavy metal ion precipitation, thereby accelerating heavy metal ion removal through coprecipitation and forming relatively stable compounds that can affect the morphology of the resulting compounds.^34^ In this study, CaCl_2_ was used as the calcium source. As shown in Fig. 5c, Cr(iii) removal was low in the absence of CaCl_2_ because Cr(iii) hydrolyzes with CO_3_^2−^ and HCO_3_^−^ in the solution, resulting in an unstable compound. Optimal Cr(iii) removal was achieved by the addition of 1.0 g CaCl_2_. Beyond this amount, Cr(iii) removal decreased, likely because excess Ca^2+^ can dehydrate or rupture the cells, thereby affecting bacterial growth and enzyme activity. Therefore, 1 g of calcium chloride was used in this study to optimize Cr(iii) removal.

The Cr(iii) concentration significantly affects its removal rate, because excessively high concentrations can be toxic to microbial strains. Fig. 5d shows the removal rate and amount of Cr(iii). For Cr(iii) concentrations of ≤1000 mg L^−1^, the removal rate exceeded 90%, achieving 98.88% and 99.08% at 500 and 1000 mg L^−1^, respectively. However, at 1500 mg L^−1^, the removal rate significantly decreased, and at 3000 and 6000 mg L^−1^, it decreased sharply to 40.66%. Despite the reduced efficiency at higher concentrations, the MICP effect was still observed across the range of 100–6000 mg L^−1^ Cr(iii). This indicates that the strain used in this study possesses high tolerance to Cr(iii), maintaining its removal activity even at elevated concentrations.

### 3.3 SEM

**Fig. 6:**
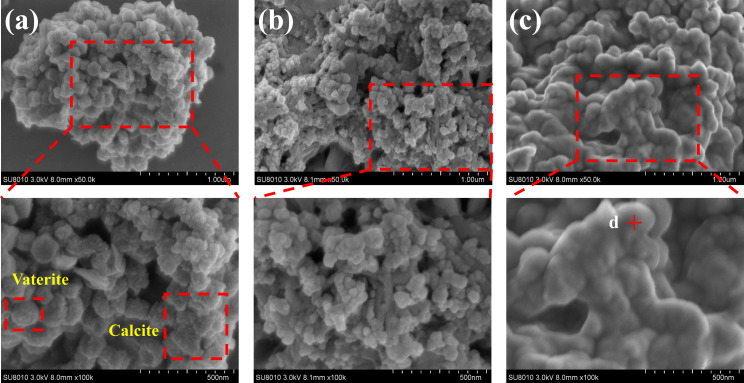
SEM images of substrates with different Cr(iii) concentrations. (a) CB + Ca; (b) CB + Ca + 1000Cr; (c) CB + Ca + 3000Cr.

**Fig. 7:**
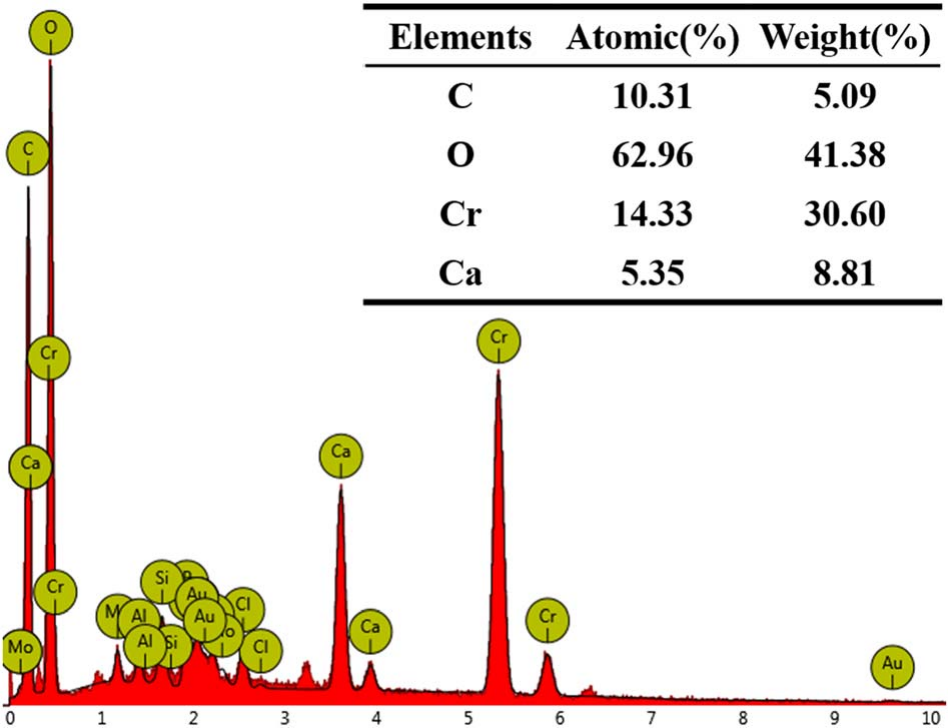
EDS of CB + Ca + 3000Cr.

The SEM images in Fig. 6a–c, magnified 50 000 and 100 000 times, illustrate the morphological changes of the MICP mineralized substrate before and after the addition of Cr(iii). In Fig. 6a, for the substrates without Cr(iii), massive and spherical particles with good dispersion and predominantly irregular precipitation were observed. The lumpy and spherical forms are identified as calcite and spherical aragonite, respectively, both forms of CaCO_3_.^35^ In Fig. 6b, the substrate with 1000 mg L^−1^ Cr(iii) shows smooth particle surfaces and a morphological shift from calcite to spherulite, with numerous particles adhering to each other. This change may be attributed to the Cr(iii) solution causing partial apoptosis of the mineralizing bacteria, creating nucleation sites for carbonate precipitation and altering the binding rate and mode, thus changing the morphology of the precipitate. Fig. 6c depicts the substrate after the addition of 3000 mg L^−1^ Cr(iii) and shows more tightly bonded carbonate mineralization, with the bonding particles mainly being spherulites. It has been suggested that calcium sources act as binders during MICP mineralization, potentially forming bioclasts that promote the formation of metal carbonates.^36^Fig. 7 presents the EDS spectrum of the substrate with 3000 mg L^−1^ Cr(iii), indicating the presence of elements such as O, Cr, Ca, and C. This suggests that carbonate compounds containing Cr and Ca were present in the mineralized substrate.

### 3.4 XRD

**Fig. 8:**
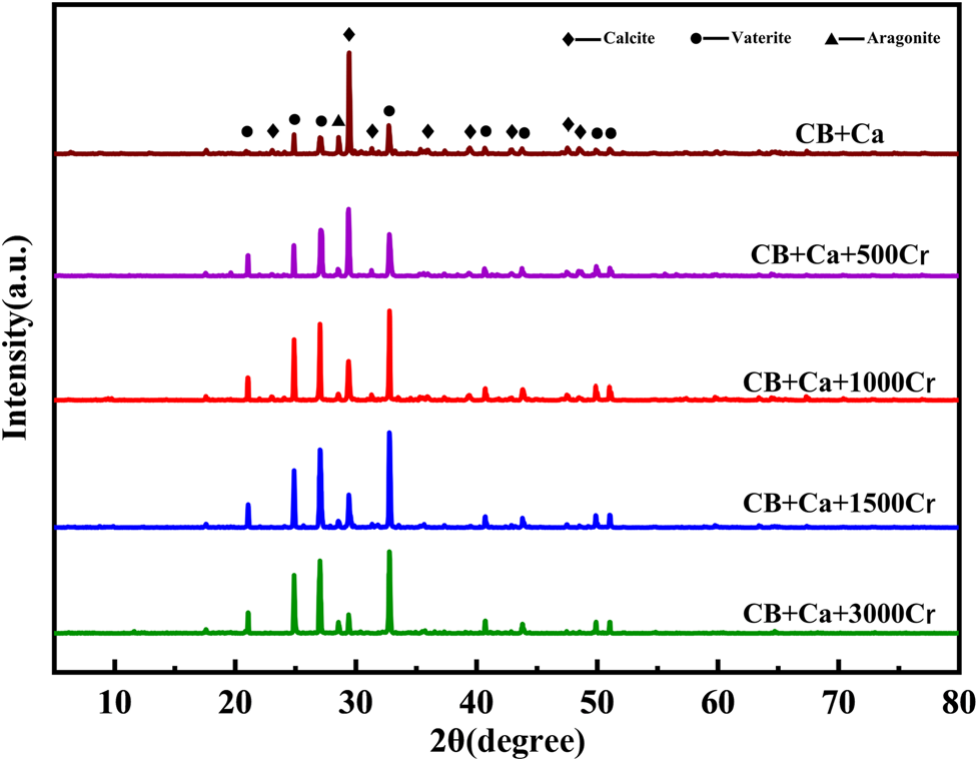
XRD patterns of substrates with different Cr(iii) concentrations. The Cr(iii) concentrations are 0, 500, 1000, 1500, and 3000 mg L^−1^.

Fig. 8 displays the results of XRD characterization of the substrates with varying Cr(iii) concentrations. By examining the intensity and position of the diffraction peaks, it was evident that the phases that precipitated from the calcium-only solution in the bacterial medium were predominantly calcite and spherulites, with calcite being the dominant phase. In addition, CaCO_3_ existed in the form of aragonite. The results indicate that the bacterial broth contains a significant amount of CO_3_^2−^, facilitated by the catalytic effect of CA on CO_2_ hydration. Compared to the XRD results without Cr(iii), the intensity of the diffraction peaks corresponding to calcite decreased with the addition of Cr(iii) solution. Specifically, the addition of 3000 mg L^−1^ Cr(iii) significantly reduced the diffraction peak intensity of calcite. Meanwhile, the intensity of the spherulite peaks increased, and after adding 1000 mg L^−1^ Cr(iii), spherulite became the dominant phase. This suggests that Cr(iii) induces the transformation of CaCO_3_ crystals from calcite to spherulite.^37^ Furthermore, no crystalline phase associated with Cr(iii) was detected in the XRD analysis after the addition of Cr(iii). However, the EDS spectrum in Fig. 7 shows the presence of Cr and Ca, indicating the formation of an amorphous material containing these elements on the substrate. Although calcite crystals can adsorb metal ions, a purely adsorptive process does not significantly alter the intensity of the CaCO_3_ diffraction peaks.^38^ Previous studies on Cr(vi) reduction demonstrated that Cr(vi) generates new compounds with CO_3_^2−^ and Ca^2+^, leading to new diffraction peaks in XRD.^39^ The absence of new peaks suggests that the valence state of Cr(iii) remained unchanged.

In summary, the XRD results show that the addition of Cr(iii) alters the structure of the CaCO_3_ crystals, causing a transformation from calcite to spherulite, which aligns with the SEM images.

### 3.5 FT-IR

**Fig. 9:**
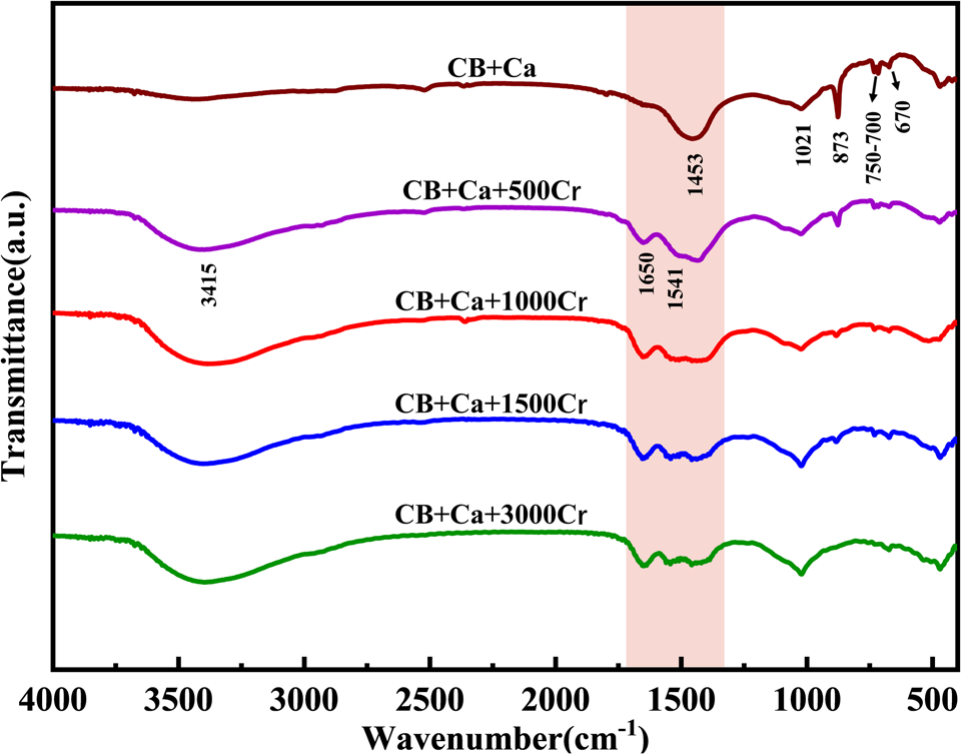
FT-IR spectra of substrates with different Cr(iii) concentrations. The Cr(iii) concentrations are 0, 500, 1000, 1500, and 3000 mg L^−1^.

Studies have shown that changes in functional groups significantly influence the carbonate mineralization process. The FT-IR results revealed several key findings (Fig. 9). The characteristic peaks of the hydroxyl (–OH) and amide groups (–NH) appeared in the 3500–3200 cm^−1^ range, with an –OH out-of-plane bending vibration peak at 670 cm^−1^,^40^ likely originating from nutrient decomposition.After adding Cr(iii), a broad, strong absorption peak at 3500–3200 cm^−1^ indicated the presence of Cr(OH)_3_ in the mineralization product. The carbonyl group (C=O) vibrational peak at 1650 cm^−1^, attributed to carboxylic acids,^41^ suggests the disruption of protein structures in peptone due to Cr(iii). The carbonate mineralization vibrational peaks are significantly affected by CO_3_^2−^, with characteristic absorption peaks at 1453 cm^−1^, 873 cm^−1^, and 750–700 cm^−1^. The broad absorption peak at 1600–1300 cm^−1^, related to the antisymmetric stretching vibration of CO_3_^2−^ due to the strong electric dipole moment of C=O in CO_3_^2−^,^42^ showed a significant weakening in intensity upon Cr(iii) addition; a new absorption peak at 1541 cm^−1^ emerged. The peaks at 873 cm^−1^ and 750–700 cm^−1^, associated with the C–O out-of-plane and in-plane bending vibrations in calcite and aragonite,^43^ respectively, diminished as the Cr(iii) concentration increased.

These changes in the CO_3_^2−^ absorption peaks suggest significant chemical bond breaking and reorganization during mineralization with the addition of Cr(iii), possibly indicating that the Ca in CaCO_3_ is replaced by Cr(iii), forming Cr(iii)-containing metal carbonates.^44^ The absorption peak at 1021 cm^−1^, corresponding to the C–O–C stretching vibration, implied that the polysaccharides in the beef paste were involved in carbonate mineralization. The increasing intensity of this peak, despite increased carbonate precipitation, suggests the formation of a new substance, likely containing a C–O–C or C–O–R functional group.

### 3.6 XPS

The elemental compositions, chemical valence states, and molecular functional group structures of the compounds on the surface of the mineralized substrate were analyzed using XPS, confirming the morphology of the compounds formed. The C 1s peak (284.8 eV) was used as a reference for the charge calibration of the other elemental peaks, and a split-peak fit was performed.

**Fig. 10:**
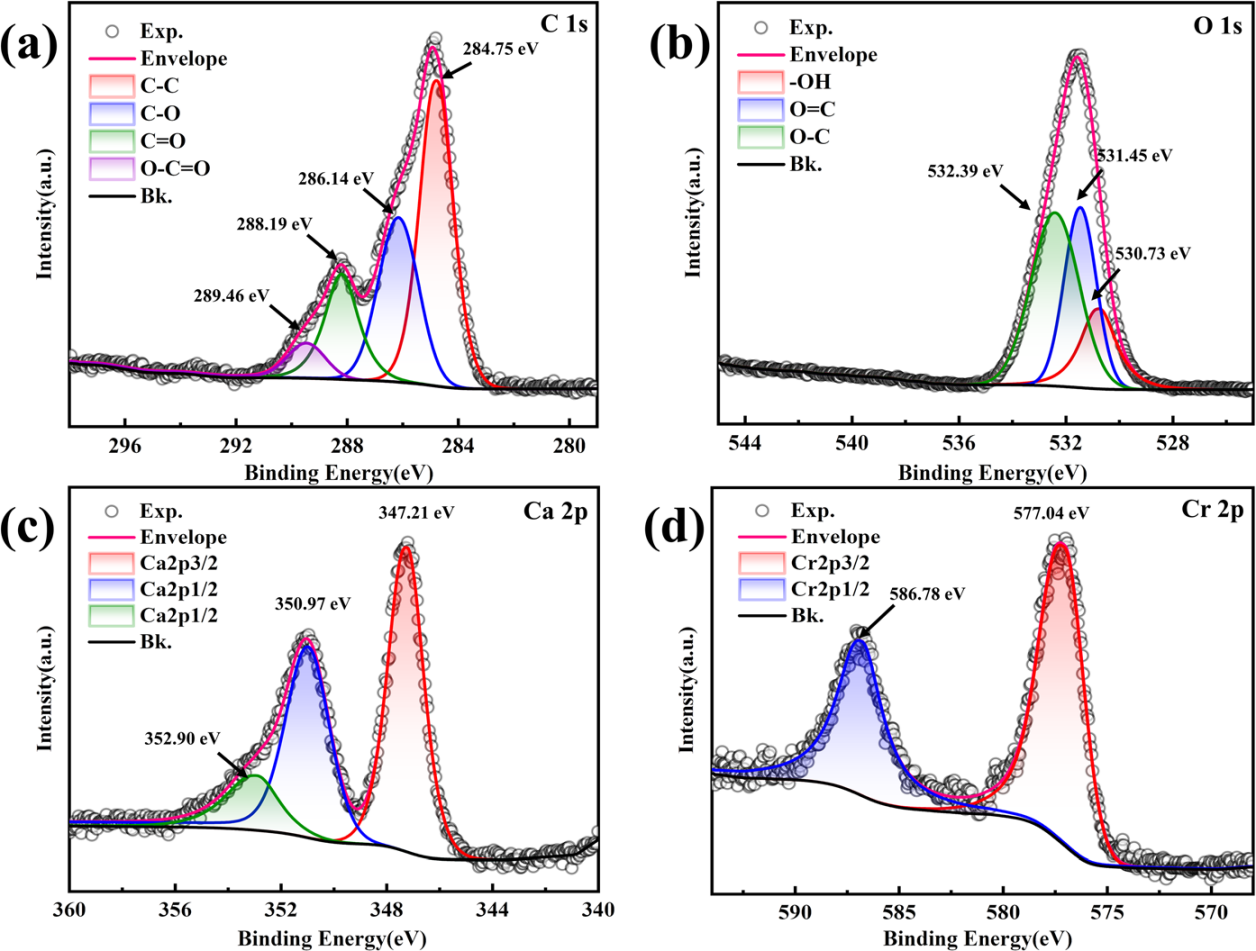
XPS spectra of the substrate at a Cr(iii) concentration of 1000 mg L^−1^. (a) C 1s; (b) O 1s; (c) Ca 2p; (d) Cr 2p.

The C 1s spectrum in Fig. 10a was fitted to four peaks attributed to C–C (284.75 eV), C–O (286.14 eV), C=O (288.19 eV), and O–C=O (289.46 eV). The presence of C–C and C=O indicated CO_3_^2−^,^45^ suggesting that the product was a carbonate compound. The C–O peak indicated ether or ether-like bonds in the compound, corresponding to the FT-IR results. The O 1s spectrum in Fig. 10b was fitted to three peaks with binding energies of 530.73 eV, 531.45 eV, and 532.39 eV, corresponding to –OH, O=C, and O–C, respectively.^46^ The O in O=C is attributed to CO_3_^2−^, further demonstrating the presence of carbonate compounds.^47^ The presence of –OH suggests hydroxides in the mineralized products or hydroxyl functional groups within the carbonate compounds, consistent with FT-IR results. The Ca 2p spectra in Fig. 10c show spin–orbit splitting peaks of 2p_3/2_ and 2p_1/2_ at 347.21 eV and 350.97 eV, respectively. These peaks are attributed to CaCO_3_,^48^ and the splitting value (Δ = *E*_2p_1/2__ − *E*_2p_3/2__) is 3.76 eV, consistent with previous studies,^49^ confirming the accuracy of the fir. Additionally, the 2p_1/2_ peaks were chemically shifted toward higher binding energy, resulting in a third peak at 352.90 eV, assigned to the metal–Ca bond,^50^ indicating a high probability of Cr–Ca bonds in the mineralized product after Cr(iii) addition. The Cr 2p spectrum in Fig. 10d shows two splitting peaks of Cr 2p_3/2_ and Cr 2p_1/2_ at 577.04 eV and 586.78 eV, respectively, characteristic of Cr(iii).^51^ No spectral peaks of Cr(vi) were observed, indicating that Cr(iii) was not oxidized to Cr(vi) in the mineralization products.

In summary, the XPS results indicated that the mineralization products contained Cr(iii)-containing carbonate compounds and hydroxides, in addition to CaCO_3_. Cr in the resulting carbonate mineralization was present as Cr(iii).

### 3.7 Mechanism of Cr(iii) removal by MICP

In general, the metal ion M forms a precipitate of metal carbonate with CO_3_^2−^; however, Cr_2_(CO_3_)_3_ is unstable because of its tendency to undergo double hydrolysis. Eqn (2)–(10) illustrate the reactions that occurred in this study: Microorganisms decompose beef paste and peptone to produce alkaline substances, such as amines. NH_3_ is hydrolyzed to produce OH^−^, causing the pH of the solution to increase. CA produced by CB promotes the hydration of CO_2_, forming HCO_3_^−^, which converts to CO_3_^2−^ as the pH increases, leading to the formation of a CaCO_3_ precipitate upon the addition of Ca^2+^.

1 M^2+^ + CO_3_^2−^ → MCO_3_↓

2 NH_3_ + H_2_O ⇌ NH_4_^+^ + OH^−^

3
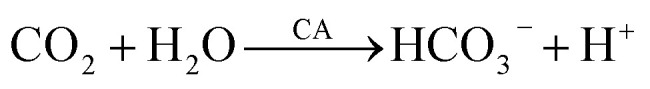

4 HCO_3_^−^ + OH^−^ → CO_3_^2−^ + H_2_O

5 Ca^2+^ + CO_3_^2−^ → CaCO_3_↓

6 2Cr^3+^ + 3CO_3_^2−^ → Cr_2_(CO_3_)_3_↓

7 Cr^3+^ + 3OH^−^ → Cr(OH)_3_↓

8 Cr^3+^ + 3OH^−^ + 2HCO_3_^−^ ⇌ CrOH(CO_3_)_2_^2−^ + 2H_2_O

9 Cr^3+^ + 4OH^−^ + CO_3_^2−^ ⇌ Cr(OH)_4_CO_3_^3−^

10 CrOH(CO_3_)_2_^2−^ + OH^−^ ⇌ Cr(OH)_4_CO_3_^3−^ + CO_3_^2−^

According to the characterization results, the internal structure of CaCO_3_ changed in the presence of Cr(iii), resulting in new Cr–Ca bonds and a morphological shift from calcite to spherulite. In an alkaline environment, Cr(iii) forms Cr(OH)_3_ precipitates. With excess HCO_3_^−^ and increasing pH, it transforms into CrOH(CO_3_)_2_^2−^ and Cr(OH)_4_CO_3_^3−^,^52^ which then form alkaline carbonate metal compounds CaCrOH(CO_3_)_2_ and Ca_3_[Cr(OH)_4_CO_3_]_2_ with Ca^2+^. These compounds are consistent with the FT-IR and XPS characterization results; therefore, it is hypothesized that the substrate contains CaCO_3_, Cr(OH)_3_, CaCrOH(CO_3_)_2_, and Ca_3_ [Cr(OH)_4_CO_3_]_2_.

## 4 Conclusions

This study experimentally verified the effectiveness of MICP in removing Cr(iii). This demonstrates that CB could achieve near-complete Cr(iii) removal under optimal conditions. The results indicated that the presence of Cr(iii) altered the CaCO_3_ crystal structure, producing Cr-containing alkaline carbonate compounds and hydroxides. This finding provides new avenues for the environmental management of Cr pollution. Although these results are significant under laboratory conditions, further optimization of process parameters and addressing variables in complex environments are necessary for practical applications. Future research should explore the application of MICP technology to various types of heavy metal pollution, optimize the conditions for large-scale operations, and investigate the integration of this technology with other treatment methods.

Despite these promising results under laboratory conditions, several challenges were encountered during the MICP process. For instance, the precipitation of Cr(iii) was affected by variations in pH and calcium concentration, which required careful optimization to ensure high removal efficiency. Additionally, the presence of Cr(iii) altered the crystal structure of CaCO_3_, which may influence the long-term stability of the precipitates and their effectiveness in large-scale applications. These challenges were addressed by adjusting experimental parameters to optimize the MICP process, ensuring maximal Cr(iii) removal while maintaining microbial growth and enzyme activity.The findings of this study are significant as they demonstrate the potential of MICP for Cr(iii) remediation, yet further optimization of process parameters and exploration of complex environmental variables are necessary for practical implementation. Future research should focus on scaling up the MICP process, optimizing conditions for field applications, and integrating this technology with other treatment methods to enhance the sustainability and efficiency of environmental remediation for heavy metals.

## Nomenclature

MICP: Microbially induced calcium carbonate precipitation
CA: Carbonic anhydrase
CB: Carbonate-mineralizing bacterium
OD_600_: Absorbance at 600 nm
AAS: Atomic absorption spectrometry
DPC: 1,5-Diphenylcarbazide
XRD: X-ray diffractometer
SEM: Scanning electron microscopy
EDS: Energy dispersive X-ray spectroscopy
FT-IR: Fourier transformation infra-red spectroscopy
XPS: X-ray photoelectron spectrometer

## Data availability

Data for this article is available at Science Data Bank at https://doi.org/10.57760/sciencedb.11756.

## Author contributions

J. Q.: conceptualization, funding acquisition, project administration, resources, writing – review and editing. H. C.: conceptualization, data curation, formal analysis, software, writing – original draft. Y. X.: investigation, methodology. F. H.: supervision, validation. F. Z.: software, visualization. W. W.: investigation, supervision.

## Conflicts of interest

There are no conflicts to declare.
